# Book Reviews

**Published:** 1919-03

**Authors:** 


### BOOK REVIEW.

### Outlines of Organotherapy.

Outlines of Organotherapy. By Henry R. Harrower, M. D., late Professor of Clinical Diagnosis, Loyola University, Chicago; Fellow of the Royal Society of Medicine; Member of the Association for the Study of Internal Secretions; etc. 110 pages, with Appendix on "Pluriglandular Therapy." Published by the Author at Glendale, California. $1.50.

The introductory chapters make a convincing plea for a more extended study of this subject. There can be no doubt that the endocrine glands need to be given more consideration in many disorders not presumed to be of obvious endocrine origin; and organotherapy is the only logical way to influence defective functioning of these vital glands. We cordially commend Doctor Harrower's book to our readers and believe that had this book been larger it would not have had as much usefulness and convenience as this concise, pocket-size "vade-mecum."

Doctor Harrower will be remembered as the author of a valuable series of papers on the internal secretions published in the *Texas Medical Journal* some years ago. This most recent of his books is the smallest, but its size is offset by the concise and boiled-down character of the contents, for, we believe, there is no such other book which has such a large amount of information on the use of glandular extracts in practice.

The arrangement is particularly good. First, the various products are classified according to their respective utility. Then each item is discussed in semi-tabular fashion under the following heads: Source and Form. Physiologic Relations and Action. Principal Therapeutic Indications. Other Minor Indications. Contraindications, Synergists. Administration and Dosage. Remarks.

### United States Army X-Ray Manual.

United States Army X-Ray Manual. Authorized by the Surgeon-General of the Army. Prepared by the Division of Roengenology. Flexible leather bound 12mo. of 506 pages. 219 illustrations. New York: Paul Hoeber, Pub., 1918.

Roengenology is a growing branch of the science of medicine.

At this time, when the attention of even the most out of the way medical practitioner is focused upon the subject, it is agreeable to see a book so compact yet so complete for use as a manual. Though intended for the medical officer near the field of action, it supplies just the information the practitioner in the country or small town needs to overcome similar handicaps. It is a book that can be recommended to all progressive practitioners.

---

### Autotherapy.

AUTOTHERAPY, by Charles H. Duncan, M. D., Discoverer of Autotherapy. Published by Dr. Charles H. Duncan, 2612 Boardway, New York City.

Dr. Duncan, taking advantage of an age long tradition that every disease carries with it its own cure and working this out in harmony with present development of Bacteriology and Immunology, has developed a system of therapy that is interesting and suggestive. It is so simple and apparently so self-evident that one wonders the ideas have been so long coming to light. The book is written in a fascinating style. Anybody reading it will certainly enjoy the book for itself and be ready to give Dr. Duncan's methods a trial. It is gratifying to see that aside from claiming priority as discoverer of the system of autotherapy, Dr. Duncan claims no special powers which are not equally available to the practitioner who will read and follow the system as explained in this book.

---

Secretary Daniels announces that he has commended fifty-two enlisted men of the navy, who, during the influenza epidemic, voluntarily submitted to experiments at the Naval Hospital at Chelsea, Mass., to aid naval doctors in the attempt to determine the cause and methods of transmission of the disease and a preventative.

Among the men of the influenza squad who were commended are: Simion G. Garrett, Oklahoma City, Okla.; R. E. Goodwin, Midlothian, Texas; Gus R. Veteo, Walter, Okla.

---

First Deaf Mute (speaking by finger signs)—What makes you stutter so?

Second Deaf Mute (speaking ditto)—I can't help it. I fell off my bicycle yesterday and sprained by first finger.—*Judge.*

